# Novel Peptidomimetic Cyclo-{E(I)-E(W)}Na (CP-88) with Hematopoietic Activity Sustained in Invasive and Oral Administration: Experimental and Preclinical Evaluation

**DOI:** 10.3390/ijms252413385

**Published:** 2024-12-13

**Authors:** Vladislav Deigin, Yulia Vinogradova, Dmitriy Vinogradov, Natalia Linkova, Anastasiia Dyatlova, Dmitrii Medvedev, Alexander Krasichkov, Victoria Polyakova

**Affiliations:** 1 Institute of Bioorganic Chemistry, Russian Academy of Sciences, Miklukho-Maklaya St., 16/10, 117997 Moscow, Russia; vdeigin@gmail.com; 2 The Department of Hospital Therapy No. 2, I.M. Sechenov First Moscow State Medical University, 8 Trubetskaya Str., 119048 Moscow, Russia; 3 Institute of Experimental Medicine, Acad. Pavlov Street, 12, 197022 St. Petersburg, Russia; 4 The Laboratory “Problems of Aging”, Belgorod National Research University, 308015 Belgorod, Russia; 5 The Department of Social Rehabilitation and Occupational Therapy, St. Petersburg Medical and Social Institute, 72A Kondratievsky St., 195271 St. Petersburg, Russia; 6 Department of Radio Engineering Systems, Electrotechnical University LETI, 5F Prof. Popova Str., 197022 St. Petersburg, Russia

**Keywords:** CP-88 peptide, peptidomimetics, cyclopeptides, hematopoiesis

## Abstract

Over the last decades, significant progress has been made in studying agonistic and antagonistic hematopoietic peptides. The main disadvantage of this class of peptides is their low stability with noninvasive administration methods, which limits the widespread use of hematopoiesis-regulated peptide drugs in medical practice. The aim of this work is to study novel peptidomimetics with hematopoietic activity sustained in invasive and oral administration. The activity of the leading compound cyclopeptide Cyclo—[Glu(Ile)-Glu(Trp)] (CP-88) was compared to that of the pharmaceutical preparation Stemokin in stimulating the population of committed colony-forming cells in intact and irradiated mice. CP-88 peptide increases the relative number of CD34+ cells in the blood and bone marrow, leading to expanded hematopoietic stem cells. CP-88 peptide, applied 48 h before bone marrow extraction, stimulates the population of committed colony-forming cells in the normal bone marrow by 33–37% above the normal level. In recipient mice injected with irradiated bone marrow, this peptide was restored practically to normal levels of colony-forming cells in a wide range of doses at intraperitoneal and oral administration. The toxicological results conclude that in humans, considering interspecies extrapolation, the CP-88 peptide can be practically safe with a single and course administration in doses of up to 100 μg/kg. The results of this investigation underscore the significant potential of CP-88 peptide as a hematopoiesis-regulated drug and instill optimism for its future application in medical practice.

## 1. Introduction

The study of hematopoiesis, a field that has significantly advanced our understanding of blood cell formation, is of immense significance. Over the past decades, it has brought about profound enlightenment, including the study of morphological data, bone marrow physiology, the biology of hematopoietic stem cells (HSCs), features of differentiation, and the maturation of all populations from the embryonic period to old age. This study is crucial for understanding blood cell formation [1].

HSCs are somatic early stem cells that give rise to several hematopoietic progenitor populations. They are the primary self-renewing source of daily hematopoiesis, a vital process that underscores the significance of their contribution to life span. This regulatory system ensures that the blood and the immune system remain independent of HSCs during the long period of human organism adaptation, providing a sense of reassurance and confidence in the robustness of our biological systems [2].

Chemokines are an essential class of proteins in the hematopoietic and immunological systems. They play a crucial role in attracting leukocytes to sites of inflammation and infection by interacting with specific receptors on the surface of target cells. Chemokines are involved in various processes of homeostasis regulation, including modulation of inflammation, hemostasis, angiogenesis, and cell proliferation. This knowledge has empowered scientists to create experimental models of maintenance, proliferation, and differentiation of human hematopoietic stem cells for further studies of hematopoiesis and the production of cells for therapy [3].

The isolation of human HSCs capable of long-term engraftment in the recipient’s body has been well-established [4].

Following HSC transplantation, hematopoiesis occurs in the recipient’s bone marrow through the interaction of donor HSCs and recipient mesenchymal cells in the bone marrow niche, which is derived from mesenchymal stem cells (MSCs) [5].

Currently, bone marrow (BM), peripheral blood (PB), and umbilical cord blood (UCB) serve as sources of stem cells for HSC transplantation [6].

In adult humans, hematopoietic stem and progenitor cells (HSPCs) reside primarily in the bone marrow (BM), a microenvironment or “niche” where HSC maintenance, self-renewal, and differentiation occur [7,8,9].

T cell development occurs primarily in the thymus. It requires migration of T cell precursors into the thymus, exploring the combination of multiple cytokines to provide a specialized microenvironment for T cell differentiation [10].

Immunomodulators are not cytokines but induce the production of hematopoietic cytokines and stimulate the regeneration of hematopoietic stem cells. Cytokine release stimulates hematopoietic progenitor and stem cells’ growth, differentiation, and proliferation. Therefore, immunomodulators are important therapeutics for radiation or cytostatic-induced injury [11].

Ideal radioprotective agents would exert a protective effect when administered before or after irradiation exposure, prevent or repair irradiation-induced tissue damage, have a rapid onset of action and long half-life, be administered orally, and be resistant to the harmful effects of irradiation and high temperatures [12].

Most protein and peptide preparations are used as injectables or infusion solutions for parenteral administration. These are inconvenient and often lead to complications and side effects [13].

Gene and cell therapy advances represent up-and-coming research fields in preventing radiation-induced damage following radiotherapy. However, it is crucial to be aware that many barriers exist to their implementation into clinical practice. The main results come from preclinical models, with very few human clinical data [14].

Short peptides are unique information molecules that have some ways of regulating many biological functions in human organisms. The DNA-peptide interaction may be the base of life. Short peptides are essential for drug creation. Drugs on the base of short peptides can have high biological activity and fewer side effects compared to small molecules [15].

Peptide’s low stability in noninvasive administration limits their wide use in medical practice [16]. Among the preferred ways of increasing peptide enzymatic stability is by modifying their linear structure by inserting a cyclic structure, which usually provides resistance to proteases’ impact. The variety of potential cyclization methods for peptide stabilization is significantly broad [17,18,19,20].

Cyclic dipeptides (diketopiperazines, DKP) are more resistant to enzyme action than linear short peptides. DKP, which contains proline, has a low hydrolysis ability and high permeability to cell membranes. These properties allow them to be used as biologically active principles of drugs [21].

A new chemical platform based on branched 2.5-piperazine-dion derivatives (2.5-diketopiperazines) has been developed to create orally available biologically active peptidomimetics [22]. This methodology has been used to obtain novel peptidomimetics with hematopoietic activity sustained in invasive and oral administration [23]. The activity of leading peptidomimetic Cyclo-{E(I)-E(W)} (CP-88) was compared to the drug Stemokin (Ile-Glu-Trp tripeptide).

Protection from hemopoiesis damage is an essential challenge of modern hematology and radiation medicine. The currently available methods for minimizing radiation injuries and stimulating post-radiation recovery of hemopoiesis can be divided into preventive and therapeutic directions. In this regard, the various approaches that will allow for decreasing the damaging effect of radiation on a population of HSCs and provide their intensive repair are under investigation. The possibility of stimulating the processes of post-radiation recovery of hemopoiesis presents a significant interest in clinical practice [11,12].

According to current findings, the loss of stem and committed hemopoietic cells, which are most sensitive to damaging factors, leads to hemopoiesis malfunctioning. The primary method for stimulating hematopoiesis is the long-term course administration of colony-stimulating (CSF)-granulocytic (G-CSF) and granulocytic-macrophage (GM-CSF) factors [24].

In our study, we developed the Ile-Glu-Trp tripeptide (Stemokin), an analog of a pharmaceutical preparation Thymogen [25]. This peptide showed a high affinity for bone marrow cells in vivo, stimulating the recovery of the bone marrow’s colony-forming unit (CFU-C) cell population after exposure to harmful treatment. It also possesses significant immuno, hemostimulating, and adjuvant effects in experimental animals and humans [26,27].

Stemokin^®^ is registered with the Ministry of Health of the Russian Federation as an immuno- and hematopoietic modulator. (Registration certificate № LSR-003016/09) [28].

We recently developed the cyclopeptide Cyclo-[Glu(Ile)-Glu(Trp)] (CP-88). The research aims to compare CP-88’s effects and Stemokin on stimulating the population of committed colony-forming cells in intact and irradiated mice both in vitro and in vivo.

## 2. Results

### 2.1. Comparison of the Biological Activity of the Peptidomimetic CP-88 and Stemokin on Intact and Irradiated Mice

The methodology described in [22] has been used to obtain novel peptidomimetics with hematopoietic activity sustained in invasive and oral administration [23].

As shown in Table 1, cyclopeptide CP-88, applied 48 h before bone marrow extraction, stimulates the committed colony-forming cells in normal bone marrow. The number of colonies statistically significantly increased after the cyclopeptide CP-88 effect, administered intraperitoneally and per os. Stemokin was not active in this test.

In the experiment with animals that received irradiated bone marrow, peptide CP-88 weakens the damaging effects in a wide range of doses of intraperitoneal and oral administration to prevent harmful radiation effects after administering irradiated in vitro bone marrow to recipient mice. Stemokin is active in a narrow range of doses only when administered intraperitoneally. Upon peroral administration, Stemokin was not active. (Table 2).

### 2.2. Studies on the Effect of CP-88 and Stemokin on Migrating CD34+ After Administration to Intact Mice and Peripheral CD117+ Blood Cells

The administration of CP-88 resulted in a statistically significant increase in the relative number of CD34+ cells in the blood and bone marrow of animals, leading to a rise in HSCs in the bone marrow and stimulating the migration of this population from the bone marrow to peripheral blood (Table 3). Along with determining the content of the CD34+ marker, a study was conducted on the drug’s effect on the appearance of a critical marker, CD117, which characterizes the level of CFU-S-8 hematopoietic and myeloid multipotent precursors.

### 2.3. Effect of Stemokin and CP-88 on NFkB Activation

**Figure 1:**
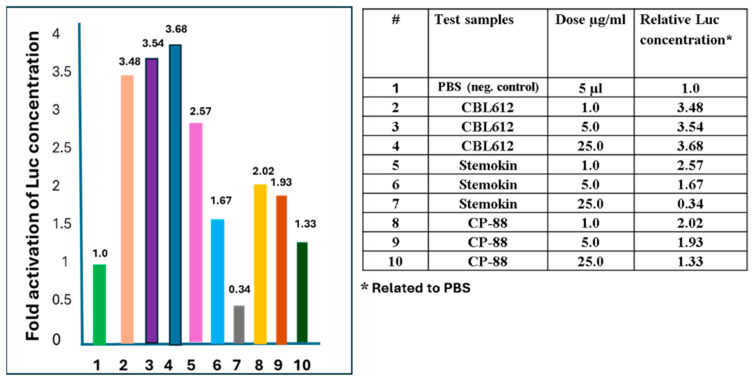
Luciferase activity in protein extracts from combined bone/bone marrow samples. CBLB612 and CP-88 treatment CBLB612 were taken as a positive control, and PBS was used as a negative control to solubilize all reagents.

To evaluate the possibility that Stemokin and CP-88 activate NF-kB nuclear activation, we compared these peptides with CBLB612, a synthetic lipopeptide JJPFGYKSHVVRDE-LXCBNONTSA-N, knowing it directly activates NF-kB nuclear translocation upon interaction with the TLR2/6 heterodimer [29]. The results are presented in Figure 1.

To evaluate the possible involvement of peptides Stemokine (3IL) and peptidomimetic CP-88 in the NF-kb pathway, NF-kB-luc reporter mice were chosen. In these mice, the luciferase expression was strictly driven by NF-kb activation in all organs and tissues.

The CBLB612 molecule was taken as a positive control, knowing it directly activates NF-kb nuclear translocation upon interaction with the TLR2/6 heterodimer. PBS was used as a negative control to solubilize all reagents. Combined bone/bone marrow samples showed the most prominent and robust NF-kB-driven luciferase activation. Thus, combining bone/bone marrow samples showed robust NF-kB-driven luciferase activation by CBLB612, Stemokin, and CP88, which showed dose-dependent activity.

### 2.4. Toxicity Study in Mice

This study of acute and chronic toxicity of CP-88 was conducted under the requirements of regulatory documents that regulate this study and are part of a complex of preclinical studies on acute and chronic toxicity that are required for the registration of drugs. Fifty-six male and fifty-six female mice, line (CBA/C57BL6) F1, were used for this study.

Acute toxicity drug tests on male and female mice administered intraperitoneally once with a volume of 0.2 mL per animal showed no toxic effects at doses of up to 10,000 µg/kg.

To determine chronic toxicity, the drug was administered intraperitoneally in a volume of 0.2 mL per animal once a day at 10 am for three months. Doses of 50 and 500 µg/kg were used, exceeding the therapeutic dose by 5 to 50 times. Animals in the control group received a placebo (distilled water).

The results of this study of chronic toxicity indicate that the test molecule, when administered intraperitoneally in doses ranging from 10 to 500 μg/kg, does not significantly affect the vital activity of animals, harm the hematopoietic system and hemostasis, have parenchymatous toxicity, or affect the functional state of the liver and kidneys.

### 2.5. Pharmacokinetics of Stemokin

The additional goal of this study was to estimate Stemokin’s pharmacokinetics using 3H-Stemokin.

The main radioactivity was accumulated in the bone marrow. This exceptional tropism to bone marrow could explain its significant hemostimulating activity (Table 4 and Table 5, line 3 underlying the extraordinary tropism of Stemokin to Bone marrow tissue).

## 3. Discussion

One of the essential goals of targeted research for many years has been the identification of substances and conditions capable of expanding HSCs ex vivo for clinical use, particularly transplantation [28].

The discovery of hematopoietic peptides from the enzymatic hydrolysis of various proteins allowed the identification of new peptide fragments that regulate hematopoiesis.

The structure of synthetic peptides used in hematology and immunology usually limits a peptide’s ability to retain some activity in the entire molecule [29]. The literature has published a few examples of favorable results in preparing hematoregulatory peptidomimetics, which converts linear peptides into cyclic analogs.

The first publication in 1990 reported hematopoietic activity isolated from mature human leukocytes, the pentapeptide pGlu-Glu-Asp-Cys-Lys (pEEDCK), HP-5). This peptide inhibits stem cell recruitment, which is a significant source of hematological complications after cytostatic tumor therapy. The disadvantage of peptide HP-5 is the oxidation of the cysteine residue, yielding a disulfide-linked dimer. A conversion of HP-5 into a peptidomimetic dimer with a suberic acid-substituted cysteine dimer stabilizes the oxidized peptide [30]. The resulting compound (pGlu-Glu-Asp)2-Sub-(Lys)_2_ (SK&F 107647) has potent hematoregulatory activity [31,32].

**Figure 2:**
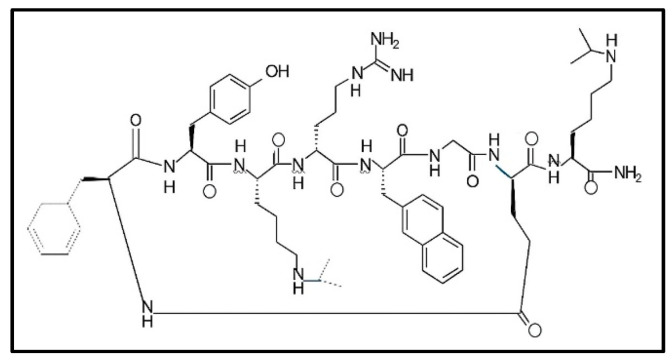
The schematic chemical structure of cyclic peptidomimetic LY2510924 [33].

In publication [33], the authors combined a medium-throughput screening and a rational design approach to create a new potent and selective CXCR4 antagonist—a cyclic peptidomimetic composed of Cyclo—[Phe-Tyr-Lys(iPr)-D-Arg-2-Nal-Gly-D-Glu]—Lys(iPr)-NH2 (LY2 510924). The structure of LY2 510924 is presented in Figure 2. The structural modeling analysis based on the published X-ray crystal structures of protein CXCR4 revealed the tentative binding conformations of peptide LY2510924, suggesting that it occupied a binding pocket and possessed ligand–receptor interactions with specific CXCR4 residues.

Considering the rapidly developing field of T cell therapy, several culture systems have been designed to support T cell development in vitro [34,35]. As a result of numerous studies, it was found that thymus peptides and their analogs can also produce a significant impact on nearly all steps of differentiation of T lymphocytes, from stem cells to effectors of cell-mediated immunity [36].

We have studied the effects of short peptides and their peptidomimetics on intact and damaged bone marrow cells to stimulate their recovery upon systemic and oral administration. The parenteral administration of Stemokin recovers the population of damaged bone marrow hemopoietic cells in mice more intensively and earlier than the colony-stimulating factors used as controls [24,37].

In our publications, we studied Stemokin and other hemoregulatory factors’ (CSF, G-CSF, and GM-CSF) ability to accelerate the restoration of bone marrow hematopoiesis after ionizing radiation. To quantitatively estimate the survival rate of the irradiated stem cells, we used their ability to form colonies in a culture or the spleen damaged by radiation. The Stemokin action was compared with the activity of the granulocyte-macrophage colony-stimulating hematopoiesis factor (GM-CSF, Leukomax) [38].

The correcting effect of Stemokin in the cytostatic cytosine-arabinoside-induced cytopenia of the hemopoietic organs has been studied. Stemokin has been shown to accelerate the restoration of the initial hemopoietic stages after cytostatic treatment, most effectively reconstituting the compartment of pluripotent precursors [24].

Given the close interaction of the immune and neuroendocrine systems, we screened a library of branched diketopiperazine derivatives in various in vitro and in vivo models [39]. In our structure-activity study, we prepared libraries of 2.5-DKP-based peptidomimetics and evaluated their biological activities (Table 6) [39].

Several cyclopeptides (A1–A5) indicated stimulatory hematopoietic activity sustained in invasive and oral administration [19]. Enantiomeric cyclopeptides (B1, B5, B6, and B9) expressed immuno and hemosuppressive activity, and peptides E1 and E3 expressed adjuvant activity [25].

Here, we investigated the biological activity of CP-88 and Stemokin in intact mice. The results showed that CP-88, when applied 48 h before bone marrow extraction, stimulated intraperitoneally and per os, increasing the committed colony-forming cells; Stemokin was inactive in this test (Table 1).

The damaging effect of ionizing radiation on hemopoiesis precursors is a limiting factor in developing radio- and chemotherapy schemes. In this regard, various approaches that decrease the harmful impact of radiation on a population of HSCs and provide their intensive repair are under investigation. The possibility of stimulating the processes of post-radiation recovery of hemopoiesis presents a permanent interest in clinical practice.

A commonly used method for identifying a population of colony-forming cells (CFU-S of exogenous spleen colonies) was used as a biological model. In this test, a suspension of bone marrow obtained from a femoral bone was administered intravenously to recipients [40]. Table 2 presents the experimental results with mice receiving irradiated in vitro bone marrow injections. Stemokin and CP-88 were administered after the injection to prevent the harmful effects of irradiation. Stemokin was active in a narrow range of doses only when administered intraperitoneally. CP-88, when administered intraperitoneally at 10 µg/kg and per os doses (10 to 2500 µg/kg), showed a stimulatory statistical effect.

One of the primary biomarkers for HSCs and HPSCs is CD34 [41]. This transmembrane phosphoglycerate is located at the cell surface in humans and various animal species. It is identified in HSCs and is an adhesion factor between cells [42]. Its expression has been detected in multiple types of cells, including hematopoietic stem/progenitor cells and MSCs. This protein is crucial in extracting and enriching HSCs for bone marrow transplantation [43]. The presence and quantity of CD34 have established the critical role of CP-88. Its use resulted in a statistically significant increase in the relative amount of CD34+ cells in animals’ blood and bone marrow. The increase in the total quantity of HSC determined by this marker stimulated their migration from the bone marrow to the peripheral blood, significantly increasing the possibility of collecting HSCs for transplantation. Along with selecting the content of the CD34+ marker, an additional study was conducted on the drug’s effect on the appearance of the critical marker CD117, which also characterizes the level of hematopoietic and myeloid multipotent precursors.

**Figure 3:**
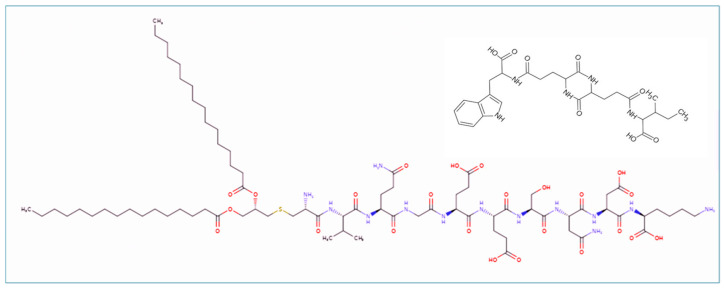
Schematic chemical structures of CBLB612 (1) and CP-88 (2), and this finding was inspiring in comparing this biological activity. CBLB612 was taken as a positive control, and PBS was used as a negative control to solubilize all reagents. Combining bone/bone marrow samples showed robust dose-dependent NF-kB-driven luciferase activation by CBLB612, Stemokin, and CP88 (Figure 1).

Several lipopeptide molecules directly activate NF-kb translocation upon interaction with the family of Toll-like receptors. CBLB502 has been shown to serve a radioprotective role in mouse and rhesus monkey models of bone marrow, gut, and reproductive damage [44,45]. This comparative study was performed with the lipopeptide formula JJPFGYKSHVVRDE-LXCBNONTSA-N (CBLB612, Figure 3), knowing its TLR2/6 heterodimer. This preparation is undergoing phase II clinical trials [27].

The toxicological test results conclude that in humans, considering interspecies extrapolation, CP-88 can be practically safe with a single and course administration in doses of up to 100 μg/kg. In the first phase of clinical trials, it is advisable to test the drug for tolerability, starting with a dose of 1 μg/kg. The dose can be increased to 10 μg/kg daily with no adverse reactions detected in the clinical results.

## 4. Materials and Methods

The procedures performed in this study followed the Guide for the Care and Use of Laboratory Animals, published by the National Institutes of Health and with the “Regulations. for Studies with Experimental Animals” (Decree of the Russian Ministry of Health from 12 August 1997, No. 755). The Institute of Ethics and the Committee of the Shemyakin-Ovchinnikov Institute of Bioorganic Chemistry approved the protocol. (Protocol No.186/2023).

### 4.1. Description of the Synthesis Process

**Figure 4:**
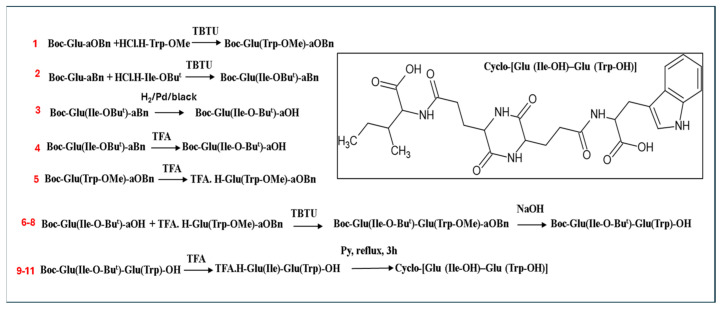
Schematic synthetic process of CP-88 preparation.

A classical synthesis method was chosen to obtain {Cyclo [Glu (Ile-OH) − Glu (TrpOH)]}e in the solution, using the maximum protection of trifunctional amino acids [18,46,47]. A schematic synthetic process of CP-88 preparation is presented in Figure 4 and Supplementary Materials.

### 4.2. Experiments in Mice

The test animals were C57B1/6 and CBAxC57Bl6 F1 female mice, 2–3 months old, 22–26 g, from the “Stolbovaya” Animal Breeding Center. All animals were in quarantine for two weeks before experiments. Only animals that appeared healthy were used for the study. A basal diet and water were available ad libitum. Environmental management was provided, including temperature regulation, ventilation and humidity regulation, lighting, bedding, and environmental enrichment. The animals were accommodated with an automated 12 h light/12 h dark cycle. Heating and cooling were electronically controlled and set to maintain the animals’ room in a temperature range between 18 °C and 22 °C.

Donor animals were intact or irradiated mice. Bone marrow from the irradiated donors was taken on day seven following irradiation. Some mice received an intraperitoneal injection of Stemokin or CP-88 in various doses (specified in Table 1 and Table 2) 48 h before bone marrow extraction. Intact donors also received peptide injections 48 h before bone marrow extraction. The bone marrow suspension (3 × 10^6^ cells per mL, 2 mL) was incubated with ^3^H-thymidine, 200 μCi, at 37 °C for 20 min, diluted to the necessary concentration, and injected into lethally irradiated recipients. The test animals were irradiated with ^60^Co gamma rays at a dose rate of 0.8 Gy per minute, bone marrow recipients with 8 Gy, and donors with 4 Gy. A suspension of bone marrow cells of intact animals was irradiated with 1 Gy 5–10 min before injection to lethally irradiated recipients.

Before drug administration to animals, a precise amount of the substance tested was dissolved in media 199 with glutamine. An aliquot was calculated based on the exact animal mass. All tested compounds were administered i/p or per os in 0.2 mL per animal using a 0.6 mm needle or catheter for oral administration. The control animals received media 199 with glutamine at the same time as the experimental group.

*Hemostimulation study.* The hemostimulating efficiency of the studied preparations was assessed by the criteria of cellularity of peripheral blood, bone marrow, and spleen in the phase of maximum bone marrow depletion (day 3) and recovery (days 7–11), as well as by the formation of splenic exocolonies.

*Peripheral blood.* The content of leukocytes, platelets, and reticulocytes was analyzed immediately after taking peripheral blood from the tail vein or the corner of the animal’s eye.

To determine the number of leukocytes, 20 μL of blood was placed in a test tube with 380 μL of 5% acetic acid. To determine the content of platelets and reticulocytes in the blood, 20 μL of blood were placed in a test tube with 3.98 mL containing 10 g of ammonium oxalate, 3 g of EDTA, and 1 mL of pharmacopeia-grade formalin in 1:1 of distilled water, with the addition of brilliant cresyl blue according to the method [48]. Blood cells were counted using light microscopy to prepare cell suspensions. Mice were sacrificed by dislocating the cervical vertebrae. The spleens and femurs were removed. The spleens were homogenized in 1–2 mL of medium 199 with glutamine. The suspension was filtered through a nylon filter; its volume was brought to 8 mL using 5% acetic acid and placed in the refrigerator until the number of splenocytes was counted using optical microscopy.

To determine the total cellularity of the bone marrow, a suspension of myelokaryocytes obtained by washing with physiological solution (1 mL) from the femur was suspended using a syringe in 5% acetic acid, and then the number of cells was counted in a Goryaev chamber on an Olympus laboratory microscope (CX21, Philippines) and the content of myelokaryocytes in the femur was determined as the volume of the calculated suspension and dilution. Cell suspensions of myelokaryocytes for subsequent administration to recipient mice were prepared, at most, 1 h before administration, storing them at the temperature of melting ice.

*Hematopoietic colony-forming stem cells.* The ability of HSCs to form colonies in the spleens of lethally irradiated mice was studied using the method of [42]. The exogenous colony formation method was used in the experiments. On the 8–9th day after the introduction of cell suspensions, the mice were sacrificed by dislocation of the cervical vertebrae; the spleens were removed fixed in Bouin’s solution, which is a mixture of a saturated aqueous solution of picric acid, formalin, and glacial acetic acid in a ratio of 15:5:1. Then, the number of macroscopically visible colonies on the surface of the spleen, the diameter of which exceeded 0.4 mm, was counted.

The arithmetic mean values and their standard errors were calculated for all the obtained variation series. To determine the significance of intergroup differences, parametric (Student’s *t*-test, Fisher’s F-test, and nonparametric Wilcoxon–Mann–Whitney, Warden, and median chi-squared tests) criteria were used and considered statistically significant when the probability integral *p-*value did not exceed 0.05. Statistical analysis was performed using the Origin 6.0 program (MicroCal Software, OriginLab Corporation, Northampton, MA, USA) [49].

*Pharmacokinetics of Stemokin* With parenteral administration of the drug, a Cmax in the blood was achieved after 5 min in the bone marrow, liver, kidneys, and lymph nodes, 30–40 min after administration. A Cmax was observed 30–40 min after administration in bone marrow, liver, kidneys, and lymph nodes. The drug’s T1/2 is 24 h and is eliminated from the body within 72 h. The pharmacokinetics of Stemokin was studied using ^3^H-Stemokin, obtained by thermal activation of gaseous tritium. 3H-Stemokin was administered intramuscularly to BDF1 female mice, and 72 h after a single dose, the levels of ^3^H-radioactivity were determined in animal organs and tissues. The main amount of radioactivity, ~80%, was excreted within 24 h after administration of ^3^H-Stemokin and was less than 5% of the administered dose (Table 4 and Table 5).

### 4.3. Effect of CP-88 and Stemokin on CD34+ Cell Migration In Vivo

The experiments on the mice demonstrated the effect of Stemokin on the content of cells carrying CD34 and markers in the blood and bone marrow. It was found that Stemokin affects the migration of CD34+ cells from the bone marrow to peripheral blood in both mice, with an elevated G-CSF background, and in control animals, this is accompanied by a change in the expression of receptors for CD29, chemokine SDF-1, and CXCR4 (CD184). CD117+ cells were determined according to [50].

Flow cytometry determined the relative number (frequency) of cells with different immunophenotypes in the peripheral blood of laboratory animals (mice). Blood samples were stained using a standard technique with combinations of monoclonal antibodies and subsequent lysis of red blood cells (lysed blood method). The study was performed using a FACS Vantage flow cytometer (Becton Dickinson Immunocytometry Systems-BDIS, Jersey City, NJ, USA) equipped with the following lasers: Spectra-Physics (488 nm), USA, and a Coherent Enterprise laser (350 nm), USA, as described in detail below.

Staining of the peripheral blood cells, spleen, and bone marrow of mice was performed as follows:

1. A total of 0.5–1 μL of rat monoclonal antibodies (Pharmingen Becton Dickinson, Jersey City, NJ, USA) against mouse CD 34-FE, mouse CD29-FE, and mouse CD184-FITC were added to labeled test tubes. To control for non-specific binding and isolate the negative region in fluorescence (based on the graphs of cell distribution by antibody binding intensity), rat IgG2a-FITC/IgG2a-FE antibodies were used at the same concentration.
2. Each test tube received 25 μL of heparinized blood and 2.5 × 10^5^ bone marrow or spleen cells. After mixing, the samples were incubated in the dark for 15–30 min.
3. Erythrocytes were lysed. The concentrated FACS-Lysing lysis solution (BDIS, USA) was diluted with distilled water ten times. The prepared solution was then added to each test tube to 500 μL, mixed, and incubated in the dark for 8 min (lysis was not performed for bone marrow and spleen).
4. Leukocytes were precipitated by centrifugation at 300× *g* for 5 min.
5. The cells were washed twice with 500 μL of PBS. The pellet was resuspended, and 100 μL of PBS was added to each tube.

The samples were analyzed on a flow cytometer within an hour of staining using the technical parameters of the device described above.

Up to 100,000 cells were analyzed in each sample, and the data on the intensity of forward and side light scattering, FITC fluorescence, and PE were saved in a file, which was processed using the CellQuestPro program (BDIS, USA).

**Figure 5:**
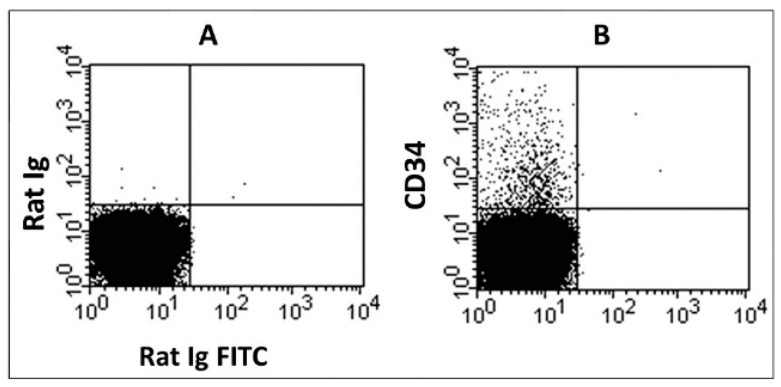
Distribution of mouse blood cells (from region R1 by the intensity of binding of rat immunoglobulins (**A**) and rat monoclonal antibodies to CD34 (**B**). CD34 cells are in the upper-left quadrant on graph (**B**).

The work used an algorithm for identifying CD34+ hematopoietic cells using the Milan protocol. The data analysis protocol is described below:

The region (Figure with low-side light scattering was isolated and stained using PE in the sample with antibodies to CD34 (B). It was assessed compared to the non-specific binding control (A). The quadrant statistics marker was placed according to the control so that at least 99% of positively stained events fell into the lower-left quadrant (Figure 5).

Data on the relative content of CD34+ cells were calculated by dividing the number of cells in the upper-left quadrant by the number of cells in region R1.

The data obtained were statistically processed using Microsoft Excel and STATISTICA 6.0 (Microcal Software, Inc., Boston, MA, USA) software packages. The distribution was considered normal at *p* > 0.05. The significance of differences in other parameters was evaluated using the Mann–Whitney U-test. The χ^2^ or Fisher criteria were used to analyze the significance of differences in the proportions of variants [51].

### 4.4. NFkB Activation by Stemokin and CP-88

The NFkB activation by Stemokin and CP-88 was performed in Cleveland Biolab (Buffalo, NY, USA).

Three mice groups (*n* = 3 per group, for each substance/dose group) were injected with three 200 µL doses (1, 5, and 25 µg/mice) subcutaneously. After injection, the mice were sacrificed at 4–5 h (which is expected to be optimal for maximal NF-kB-driven luciferase expression). Eleven organs, including the bladder, spleen, mesenteric lymph nodes, ileum, colon, kidney, liver, lung, bones, blood, and thymus, were collected and frozen on dry ice for further processing and protein extraction.

All organs were homogenized on dry ice to a powder condition using a pulverizer “pistol device” (Biospec Products, Inc., Bartlesville, OK, 74005, USA) for 2–3 consecutive “shots.”

For protein extraction, two volumes of lysis buffer (Promega #E397A, Promega Corp, Medison, WI, 53711, USA) were added to the tube, as well as 1 volume of ZrSiO beads (0.5 mm beads, Next Advance #ZSB05, Next Advance, Inc., Averill Park, NY, 12018, USA). The samples were homogenized and lysed in a bullet blender (Bullet Blender Blue, Model BBX24B, Next Advance) at 8 for 3 × 15 sec (mixed by hand between rounds). The Protease Inhibitor Cocktail (Sigma # P8340, Lot # 083M4020V) was added 1:100 to the lysis buffer just before being added to the samples.

Lysates were cleared by centrifugation for 30 min at 13,000 rpm at 40 °C and transferred to clean tubes. They were stored at −80 °C before the protein concentration and luciferase activity measurements.

The protein concentration was measured for each sample using the PierceTM BCA protein assay kit (Thermo Scientific, #23227, Pierce Biotechnology, Rockford, IL, 61105, USA), following the recommended instructions.

Following the instructions, the luciferase activity was measured using the Bright-Glo Luciferase assay system (Promega, E2610). In the first step, the exact amount of lysate from each sample (5 µL) was used. The RLU was calculated per mg of protein using the measured protein concentration.

CBLB612 was taken as a positive control, and PBS was used as a negative control to solubilize all reagents. The protein concentration was re-measured for the bone/bone marrow samples at the next step. The number of samples for the luciferase assay was re-adjusted to have the same amount of protein for each sample (the protein concentration was equalized to sample CBLB62, which contained less concentrated protein lysate—injection with 25 µg of CBLB62; average dilution of the samples—6 folds). The experimental data were processed using ANOVA analysis.

### 4.5. Acute and Chronic Toxicity of CP-88

To determine acute toxicity, peptide was administered to male and female mice (CBA/C57BL6) F1 (56 male and 56 female mice). The animals were obtained from the Scientific and Production Enterprise “Stolbovaya” Laboratory Animal Nursery, Moscow Region, at the age of 4 weeks. Upon receipt, an external examination of the animal’s condition was conducted.

The drug was administered in the form of aqueous solutions intraperitoneally in doses of 500, 1000, and 10,000 μg/kg in a volume of 0.2 mL per mouse, which exceeded the effective doses of the drug by 50 to 1000 times. Animals of the same batch, injected with distilled water in a volume of 0.2 mL per animal (control substance), were used as a biological control.

The results of the drug’s acute toxicity and tolerability tests, conducted on mice, allow us to conclude that the drug does not have toxic properties when administered parenterally at doses of up to 10,000 μg/kg.

To determine chronic toxicity, the drug was administered intraperitoneally in a volume of 0.2 mL per animal once a day at 10 am for three months. The animals were weighed immediately before the drug was administered. Doses of 50 and 500 μg/kg were used, exceeding the therapeutic dose by 5 to 50 times. The animals in the control group received a placebo (distilled water). The results of the study of chronic toxicity indicate that the test peptide, when administered intraperitoneally in doses ranging from 10 to 500 μg/kg, does not cause significant deviations in the vital activity of animals, does not hurt the hematopoietic system and hemostasis, does not have parenchymatous toxicity, and does not affect the functional state of the liver and kidneys. The results of the autopsy, as well as the histomorphology study of internal organs, biochemical blood analysis, and assessment of the metabolic state of the liver, also indicate that the drug does not cause disturbances of the main metabolic processes in the body and does not affect the function of endocrine organs and electrolyte balance.

## 5. Conclusions

The transformation of linear peptides into stabilized peptidomimetics by converting them into 2.5-diketopiperazine provides enzymatic stabilization of the compound without loss of functional properties, showing their potential oral applicability. 

It has been proven that Stemokin injections accelerate the restoration of the initial stages of hematopoiesis after irradiation and treatment with cytostatics. The oral administration of CP-88 revealed that the drug has all the properties of Stemokin and a new, unique activity—the ability to stimulate the accumulation of intact bone marrow cells. Experimental data and toxicology studies show that peptidomimetic CP-88 is a promising drug for stimulating hematopoiesis for bone marrow reparation after damaging effects. The next development step is to continue the experimental and preclinical development of CP-88 and advance it to clinical trials.

## Figures and Tables

**Table 1:** Stimulatory effect of peptides on the bone marrow of intact mice.

Preparation	Administration	Dose, µg/kg	Number of Colonies /10^5^ Cells	% To Control
Control	-	-	14.5 ± 1.6	100
Stemokin	I/P	100	14.7 ± 0.7	100
	per os	100	14.3 ± 1.8	100
CP-88	I/P	100	19.9 ± 1.3 *	137 *
	per os	1000	19.3 ± 1.3 *	133 *

*—*p* < 0.05 compared to control.

**Table 2:** Effect of CP-88 and Stemokin on the formation of exogenous colonies by bone marrow irradiated in vitro (1 Gy).

№	1 Gy	Dose µg/kg CP-88	Administration	Number of Colonies /10^5^ Cells
1	Control	-	-	14.9 ± 0.5
2	+	-	-	7.4 ± 0.4 **
3	+	100	IP	11.9 ± 0.4 *
4	+	10	per os	11.6 ± 0.8 *
5	+	500	per os	14.5 ± 0.6 *
6	+	1000	per os	10.7 ± 0.6 *
7	+	2500	per os	12.4 ± 1.1 *
**№**	**1 Gy**	**Dose µg/kg** **Stemokin**	**Administration**	**Number of** **Colonies** **/10^5^ Cells**
1	Control	-	-	10.6 ± 0.4
2	+	-	-	5.6 ± 0.5 **
3	+	2.5	IP	6.0 ± 0.7
4	+	10	IP	8.7 ± 0.4 *
5	+	10	IP	8.2 ± 0.7 *
6	+	10	IP	10.6 ± 0.8
7	+	25	IP	7.6 ± 0.4 *
8	+	50	IP	7.1 ± 0.5 *
9	+	10	per os	5.4 ± 0.5

*—*p* < 0.05 compared to control; ** *p* < 0.05—compared to irradiated animals.

**Table 3:** Effect of Stemokin and peptide CP-88 on the relative content of CFU-S-8 and CD34+ hematopoietic cells in intact mice bone marrow and peripheral blood.

Group	Peripheral Blood (CD117+)	Bone Marrow (CD34+)
	Average CFU-S-8	% CD34+ Cells
Control	10.5 ± 0.3 (20)	0.4 ± 0.09 (10)
Stemokin	14.07 ± 0.6 * (20)	0.8 ± 0.05 * (10)
CP-88	15.2 ± 0.7 * (20)	0.7 ± 0.05 * (10)

*—*p;* < 0.05 compared to control; number of mice is shown in brackets.

**Table 4:** The pharmacokinetics of Stemokin in mice using 3H-Stemokin content in various tissues.

№	Organ, Tissue	^3^H-S Content (ng/g Wet Tissue)							
		Sampling Time After Injection of 3H-T, Hours							
		0.083	0.25	0.5	1.0	3.0	5.0	24.0	72.0
1	Blood	3.3	4.0	3.9	1.8	1.2	1.0	0.7	0.5
2	Plasma *	4.7	5.7	5.2	1.9	1.1	1.0	0.8	0.4
3	Bone marrow	27.5	42.5	45.0	32.5	25.0	22.5	22.5	15.0
4	Kidneys	10.4	17.0	20.4	10.4	5.8	4.8	2.7	1.3
5	Liver	3.7	7.0	8.2	4.5	2.5	2.1	1.2	0.6
6	Spleen	1.7	2.3	3.0	2.1	1.5	1.3	0.9	0.7
7	Thymus	1.4	2.5	2.4	1.8	1.2	1.2	0.9	0.6
8	Lymph nodes	4.8	5.4	3.4	2.1	1.1	1.1	0.7	0.5
9	Brain	0.7	1.0	1.1	1.1	0.8	0.8	0.7	0.5
10	Plasma of 1 mL blood, ng	2.8	3.4	3.1	1.1	0.7	0.6	0.5	0.3
11	E Elements of 1 mL blood, ng	0.5	0.6	0.8	0.7	0.5	0.4	0.2	0.2

*—ng/mL.

**Table 5:** The pharmacokinetic parameters of 3H-Stemokin in mice.

№	Organ, Tissue	t (Cmax), Hours	Cmax (ng/g)	Cmax (Organ)/Cmax Blood	C 24 h, % of C max	AUC 72 h (ng hour/g)	AUC 72 h Organ/AUC 72 h Blood	MRT, Hours
1	Blood	0.25	4	1	17.5	53.2	1	28
2	Plasma	0.25	5.7 *	1.4	14	55 **	1	25.4
3	Bone marrow	0.5	45	11.2	50	1469.7	27.6	30.9
4	Kidneys	0.5	20.4	5.1	13.2	208.7	3.9	22.6
5	Liver	0.5	8.2	2	14.6	92.1	1.7	23.2
6	Spleen	0.5	3	0.75	30	68	1.3	29.6
7	Thymus	0.25	2.5	0.6	36	63.3	1.2	28.9
8	Lymph nodes	0.25	5.4	1.3	13	54.6	1	27.4
9	Brain	0.5	1.1	0.3	63.6	47.5	0.9	31

*—*p* < 0.05 ng/mL, **—*p* < 0.01—ng/h/mL.

**Table 6:** Examples of the library of cyclopeptides used for screening.

Peptide Code	Tested Molecule	R^1^
A1	c[Asp(IleNH_2_)Glu(Trp)]	-(CH)CO[NHCH(CONH_2_)-(CH_3_)CH_2_CH_3_]
A3	c[Asp(Ile)Glu(Trp)]	-(CH)CO[NHCH(COOH)-(CH_3_)CH_2_CH_3_]
A4	c[Glu(Ile)Glu(Trp)]	-(CH)_2_CO[NHCH(COOH)(CH_3_)CH_2_CH_3_]
A5 A6 A10	c[AlaGlu(Trp)] c[ArgGlu(Trp)] c[LeuGlu(Trp)]	-CH_3_ -NHCH(CH_2_)_3_[NHCNH_2_=NH] -CH_2_CH(CH_3_)_2_
A11 B1 B5 B6	c[LysGlu(Trp)] c[DAlaDGlu(DTrp)] c[DLysDGlu(DTrp)] c[DPheDGlu(DTrp)]	-CH(CH_3_)-NH_2_ -CH_3_ -CH(CH_3_)-NH_2_ -CH-CH_2_-Ph
B9	c[DTyrDGlu(DTrp)]	-CH-CH_2_-Ph(OH)
B10	c[DValDGlu(DTrp)]	-CH(CH_3_)_2_
C1 C2 E1	c[AlaGlu(Tyr)] c[LeuGlu(Tyr)] cyclo[L-Lys(N-acetyl-Glucosamine-N-acetyl-muramyl)-L-Glu(L-Trp)]	-CH_3_ -CH_2_CH(CH_3_)_2_ -CH(CH_3_)-NH(N-acetyl-Glucosamine-N-acetyl-muramyl)
E2	cyclo[D-Lys(Palmitoyl)-D-Glu(D-Trp)-OH]	-CH(CH_3_)-NH(Palmitoyl)
E3	cyclo[Lys(Palmitoyl)-Glu(D-Trp)]	-CH(CH_3_)-NH(Palmitoyl)
E5	cyclo[D-Lys(N-acetyl-Glucosamine-N-acetyl-muramyl)-d-Glu(D-Trp)]	-CH(CH_3_)-NH(N-acetyl-Glucosamine-N-acetyl-muramyl)

## Data Availability

The data presented in this study are available upon request from the corresponding author.

## Supplementary Materials

The following supporting information can be downloaded at: https://www.mdpi.com/article/10.3390/ijms252413385/s1.
