# Supplementary material for: Novel Peptidomimetic Cyclo-{E(I)-E(W)}Na (CP-88) with Hematopoietic Activity Sustained in Invasive and Oral Administration: Experimental and Preclinical Evaluation
Source: Int J Mol Sci. 2024 Dec 13;25(24):13385. doi: 10.3390/ijms252413385 (PMC11679558; doi:10.3390/ijms252413385)
Supplement: Supplementary file 1 [file ijms-25-13385-s001.zip › ijms-3347922-supplementary.pdf]

Supplemental materials.

The synthesis of {Cyclo [Glu (Ile-OH) – Glu (TrpOH)]}peptidomimetic.

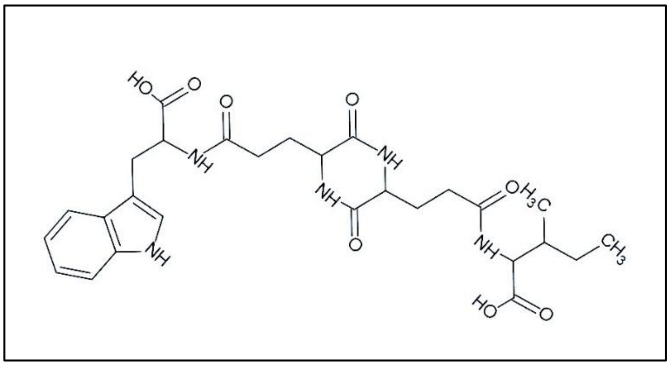

Chemical structure of {Cyclo [Glu (Ile-OH) – Glu (TrpOH)]}

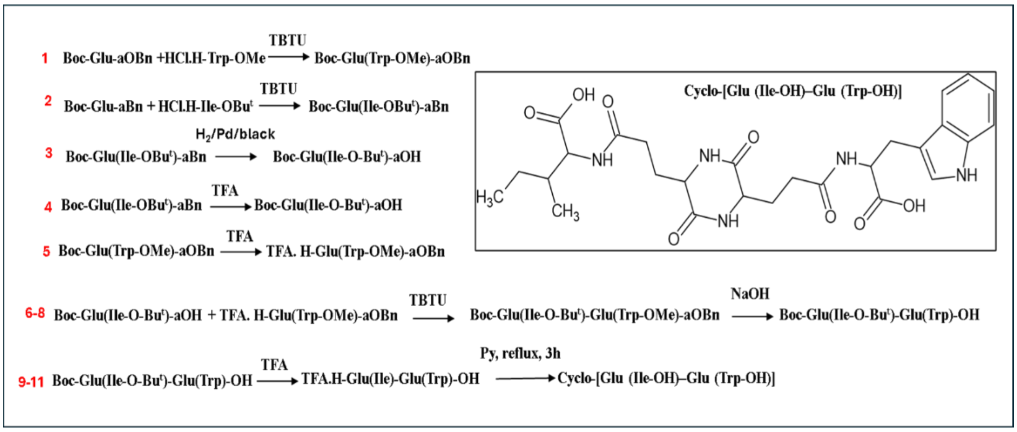

Schematic synthetic process of CP-88 preparation.

Description of the synthesis process.

A classical synthesis method was chosen to obtain {Cyclo [Glu (Ile-OH) – Glu (TrpOH)]}e in the solution, using the maximum protection of trifunctional amino acids.

Step 1. Obtaining - methyl ester of N - tert-butyloxycarbonyl L-glutamyl (benzyl) - tryptophan.
N-α-t-butyloxycarbonyl-L-glutamic acid (alpha-benzyl ester) reacts with tryptophan methyl ester hydrochloride in 1,4-dioxane at room temperature in the presence of 4-N-methylmorpholine and N, N, N', N'-tetramethyl-O-(benzotriazol-1-yl)uronium tetrafluoroborate (TBTU).

Methyl ester of N - tert - butyloxycarbonyl L-glutamyl (benzyl) - tryptophan is formed. The liquid obtained from the reaction is evaporated on a rotary evaporator under reduced pressure and a temperature not exceeding 40° until almost complete evaporation of the solvent and an oily residue is obtained. The residue is dissolved in ethyl acetate.

The resulting solution is successively washed with several portions of sulfuric acid solution, several portions of sodium bicarbonate solution, and then with water (the washing liquid is discarded). The washed solution is dried over anhydrous sodium sulfate and filtered through a glass filter.

The filtered solution is evaporated to a small volume on a rotary evaporator at a temperature not exceeding 40°. A certain amount of ethyl ester and n-hexane are added to the residue. Methyl ester of N-tert-butyloxycarbonyl L-glutamyl (a-benzyl) - tryptophan crystallizes from the resulting mixture.
The crystallization product obtained in Step 1.2 is separated by filtration through a glass filter and washed on the filter with several portions of a mixture of ethyl ester and n-hexane. The washed crystals of methyl ester of N-tert-butyloxycarbonyl L-glutamyl (a-benzyl) - tryptophan are dried in a vacuum at room temperature for several hours.

Step 2. Obtaining - tert-butyl ester of N-tert-butyloxycarbonyl L-glutamyl(benzyl)-isoleucine.
N-α-t-butyloxycarbonyl-L-glutamic acid (a-benzyl ester) reacts with hydrochloride of tert.butyl ester of isoleucine in 1,4-dioxane at room temperature in the presence of 4-N-methylmorpholine and N,N,N',N'-tetramethyl-O-(benzotriazol-1-yl)uronium tetrafluoroborate (TBTU).
Tert-butyl ester of N-tert-butyloxycarbonyl L-glutamyl(benzyl)-isoleucine is formed. The liquid obtained from the reaction is evaporated on a rotary evaporator under reduced pressure and a temperature not exceeding 40° until almost complete evaporation of the solvent and an oily residue. The residue is dissolved in ethyl acetate.
The resulting solution is successively washed with several portions of sulfuric acid solution, several portions of sodium bicarbonate solution, and then with water (discard the washing liquid). The washed solution is dried over anhydrous sodium sulfate and filtered through a glass filter.
The filtered solution is evaporated to a small volume on a rotary evaporator at a temperature not exceeding 40°. A certain amount of ethyl ester and n-hexane are added to the residue. Tert-butyl ester of N-tert-butyloxycarbonyl L-glutamyl (benzyl) isoleucine crystallizes from the resulting mixture.
The crystallization product obtained in Step 2 is separated by filtration through a glass filter and washed on the filter with several portions of a mixture of ethyl ester and n-hexane (discard the washing liquid). Washed crystals of a tert-butyl ester of N-tert-butyloxycarbonyl L-glutamyl(benzyl)-isoleucine are dried in a vacuum at room temperature for several hours.

Step 3. Obtaining Boc - Glu (Ile-OtBu) –OH
Dissolve the tert-butyl ester of N—tert-butyloxycarbonyl L-glutamyl (benzyl)—isoleucine in 200 ml of ethanol, adding palladium black (1:10). Pass a stream of hydrogen through the solution for 3 hours. TLC monitors the progress of the reaction in the system Chloroform: ethyl acetate: methanol = 6:3:1. After the reaction, the solution evaporates to oil. The resulting product is transferred to Step 4.

Step 4. Obtaining methyl ester of N - tert - butyloxycarbonyl L-glutamyl (L-isoleucyl -tert-butyl ester) - L-glutamyl (alpha-benzyl) – tryptophan.
The intermediate product obtained in Step 1 is dissolved in a 70% aqueous trifluoroacetic acid solution. The solution is thoroughly mixed for 40 minutes and then evaporated to a small volume using a rotary evaporator under reduced pressure and a temperature not exceeding 40°. Ester is added to the resulting oily residue. An oily residue of methyl ester - L-glutamyl (alpha-

benzyl) - tryptophan falls out. Dissolve tert-butyl ester of N-tert-butyloxycarbonyl L-gamma-glutamyl – isoleucine in 1,4-dioxane at room temperature in the presence of 4-N-methylmorpholine and N, N, N', N'-tetramethyl-O-(benzotriazol-1-yl)uronium tetrafluoroborate (TBTU). After 2 minutes, add a solution of methyl ester of - L-glutamyl (alpha-benzyl) – tryptophan and 4-N-methylmorpholine in a mixture of DMF and dioxane (50%). The mixture is stirred for 2 hours. Methyl ester of N-tert-butyloxycarbonyl L-glutamyl (L-isoleucyl -tert-butylyl ester) - L-glutamyl (alpha-benzyl) – tryptophan is formed. The liquid obtained from the reaction is evaporated on a rotary evaporator at reduced pressure and a temperature not exceeding 40° until almost complete evaporation of the solvent and an oily residue. The residue is dissolved in ethyl acetate. The resulting solution is successively washed with several portions of a sulfuric acid solution, several portions of a sodium bicarbonate solution, and then with water (the washing liquid is discarded). The washed solution is dried over anhydrous sodium sulfate and filtered through a glass filter. The filtered solution is evaporated to a small volume on a rotary evaporator at a temperature not exceeding 40°. A certain amount of ethyl ester and n-hexane are added to the residue. Methyl ester of N - tert - butyloxycarbonyl L-glutamyl (L-isoleucyl -tert-butylyl ester) - L-glutamyl (alpha-benzyl) - tryptophan crystallizes from the resulting mixture. The crystallization product obtained in Step 2 is separated by filtration through a glass filter and washed on the filter with several portions of a mixture of ethyl ester and n-hexane (the washing liquid is discarded). The washed crystals of methyl ester of N-tert-butyloxycarbonyl-L-glutamyl (L-isoleucyl-tert-butylyl ester) - L-glutamyl (alpha-benzyl) - tryptophan is dried in a vacuum at room temperature for 4 hours.

Step 5. Obtaining N - tert -butyloxycarbonyl L-glutamyl (L-isoleucyl -tert-butylyl ester) - L-(tryptophanyl) - glutamic acid.
Dissolve methyl ester of N - tert –butyloxycarbonyl-L-glutamyl (L-isoleucyl-tert-butylyl ester) - L-glutamyl (alpha-benzyl)- tryptophan in 100 ml of ethanol, add the calculated amount of 4N NaOH. Stir at room temperature for 1 hour. Control - TLC in the CHEM system (Chloroform: ethyl acetate: methanol = 6:3:1). Evaporate the alcohol on a rotary evaporator at 40 ° C. Add H_2O and acidify to pH = 3 with a 5% sulfuric acid solution. Extract with ethyl acetate. The resulting organic solution is successively washed with several portions of sulfuric acid solution and water (the washing liquid is discarded). The washed solution is dried over anhydrous sodium sulfate and filtered through a glass filter. The filtered solution is evaporated to oil on a rotary evaporator at a temperature not exceeding 40°. Dissolve in a minimum volume of ethyl acetate and precipitate from ethyl acetate with hexane. The resulting precipitate is left in the refrigerator overnight. Then, it was filtered, washed with ester and hexane, and dried in air.

Step 6. Obtaining trifluoroacetate L-glutamyl (L- isoleucyl -) - L- (tryptophanyl) - glutamic acid (TFU.N- Glu (Ile-OH) - Glu (TrpOH) - OH). Dissolve N-tert-butyloxycarbonyl L-glutamyl (L-isoleucyl-tert-butylyl ester) - L- (tryptophanyl) - glutamic acid in 80% TFA and stir for 3 hours. The reaction progress is monitored by TLC (CHEM + 0.1% AcOH) and analytical HPLC. Upon completion of the reaction, evaporate the solution in a vacuum to an oily state and add ester. The resulting precipitate is filtered, washed with ester, and dried in air. The reaction product is transferred to Step 7.

Step 7. Obtaining cyclo-{glutamyl(isoleucyl)-(tryptophanyl)glutamic acid} - {Cyclo [Glu (Ile-OH) – Glu (TrpOH)]}

Dissolve trifluoroacetate L-glutamyl(L-isoleucyl—)—L-(tryptophanyl)—glutamic acid in pyridine and reflux for 3 hours. The reaction progress is monitored by TLC (CHEM + 0.1% AcOH) and analytical HPLC. After the reaction, the solvent is evaporated in a vacuum, the reaction product is precipitated with ester, and the precipitate is filtered off and dried in air.

Step 8. Chromatographic purification.

Cyclotetrapeptide cyclo–{L-Glu(L-Ile)-L-Glu(L-Trp)}, obtained after complete deblocking, was purified on a preparative column (50x300 mm) with silica gel C18.

Peptide	MW	MS^+	Purity
**Cyclo-[Glu(Ile-OH)-Glu(TrpOH)]**	557.6	558,3	99.3

Analytical data.

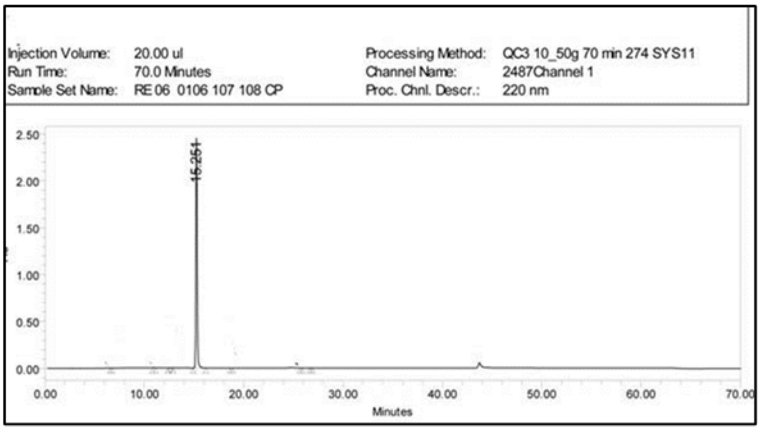

Figure 1. HPLC chromatogram of cyclo tetrapeptide cyclo – {L-Glu(L-Ile) – L-Glu(Trp)}.

Waters chromatographic system. Column 4.6x25 cm, 5μ, Symmetry C18.

Gradient 1% per minute from 10 to 70% acetonitrile in 0.1 M triethylammonium phosphate buffer, pH 3.9. UV detection at 220 nm.
